# Antibiotic Resistance Awareness and Prescribing Behavior Among General Practitioners in Kazakhstan, Kyrgyzstan, Uzbekistan, and Tajikistan

**DOI:** 10.3390/antibiotics15030309

**Published:** 2026-03-18

**Authors:** Yuliya Semenova, Kamila Akhmetova, Shakhnoza Rakhmatullaeva, Makhbuba Muminova, Dilafruz Fakhriddinova, Kenesh Dzhusupov, Asel Kanymetova, Damira Ashyralieva, Mukhabbat Saidova, Shakhlo Yakubova, Lyudmila Pivina, Zaituna Khismetova

**Affiliations:** 1 Department of Surgery, School of Medicine, Nazarbayev University, Astana 010000, Kazakhstan; yuliya.semenova@nu.edu.kz; 2 Department of Public Health and Management, Astana Medical University, Astana 010000, Kazakhstan; 3 Department of Infectious Diseases, Tashkent State Medical University, Tashkent 100109, Uzbekistan; sh.rakhmatullaeva@tashmeduni.uz (S.R.); makhbuba.muminova@tashmeduni.uz (M.M.); d.fakhriddinova@tashmeduni.uz (D.F.); 4 Department of Public Health, International Higher School of Medicine, Bishkek 720054, Kyrgyzstan; k.dzhusupov@ism.edu.kg; 5 Scientific and Practical Center for Infection Control at the National Institute of Public Health, Bishkek 720014, Kyrgyzstan; asel.kana09@mail.ru (A.K.); ashyr19611411@gmail.com (D.A.); 6 Department of Pharmaceutical Chemistry and Pharmacy Management and Economics, Tajik National University, Dushanbe 734025, Tajikistan; sald_mouhabbat@mail.ru (M.S.); shakhlo.yakubova.99tnu@mail.ru (S.Y.); 7 Department of Emergency Medicine, Semey Medical University, Semey 071407, Kazakhstan; lyudmila.pivina@smu.edu.kz; 8 Department of Public Health, Semey Medical University, Semey 071407, Kazakhstan; zaituna.khismetova@smu.edu.kz

**Keywords:** antimicrobial resistance awareness, general practitioners, antibiotic prescribing behavior, Central Asia

## Abstract

Background/Objectives: Despite a wide range of international studies examining antibiotic prescribing practices among physicians, research from Central Asia remains scarce. To address this gap, the present study aimed to investigate antibiotic resistance awareness and prescribing practices among general practitioners (GPs) in Kazakhstan, Kyrgyzstan, Uzbekistan, and Tajikistan. Methods: The online questionnaire was completed by 1231 GPs, including 469 from Kazakhstan, 274 from Kyrgyzstan, 369 from Uzbekistan, and 119 from Tajikistan. Results: Most physicians (71.1%) acknowledged that their antibiotic prescribing behavior influences the development of antibiotic resistance in their regions. More than half reported discussing antibiotic resistance with their patients often or very often. However, the strategy of delayed antibiotic prescribing was unknown to 27.1% of GPs. Factors associated with good knowledge of indications for antibiotic prescribing included female sex, older age, working in Uzbekistan, practicing in urban areas, seeing 20 or more patients per day, and use of practice guidelines. Clinical practice guidelines were the most frequently reported source of current information on antibiotic therapy and resistance (20.4%), followed by continuing professional education (15.9%) and textbooks (14.1%). The vast majority of GPs (94.4%) indicated a need for additional information resources to support more rational antibiotic prescribing. The most commonly cited needs were higher-quality clinical practice guidelines (22.5%) and better access to existing guidelines (17.7%). Conclusions: These findings suggest that, despite generally high awareness of antibiotic resistance, important knowledge gaps remain among GPs in Central Asia. Strengthening access to clinical guidelines and continuing professional education may support more rational antibiotic prescribing.

## 1. Introduction

Antimicrobial resistance (AMR) is a growing global public health concern. According to the Global Burden of Disease study, under current trends, approximately 1.91 million deaths per year are projected to be directly attributable to bacterial AMR by 2050 [1]. The misuse and overuse of antibiotics across human and animal healthcare, as well as in agriculture and aquaculture, are major drivers of the emergence and spread of AMR. To address this issue, the One Health approach has been developed, emphasizing the interconnectedness of human, animal, and environmental health and fostering collaboration across these sectors to prevent and control AMR [2].

Within the human healthcare sector, several factors contribute to the inappropriate use of antibiotics. Public misconceptions that antibiotics are effective for a wider range of conditions than they actually are, along with the perception that these drugs are inherently safe, increase demand [3]. In countries where over-the-counter (OTC) sales are not strictly regulated, this demand significantly drives community antibiotic consumption, as pharmacists may dispense antibiotics for profit [4]. In countries with effective OTC control, patients must consult a physician to obtain a prescription. However, faced with clinical uncertainty or patient pressure, physicians may over-prescribe antibiotics [5]. Therefore, it is essential for physicians to recognize the challenges posed by AMR and adhere to ethical, evidence-based prescribing practices [6].

General practitioners (GPs) form the backbone of the primary healthcare workforce in many countries. They are often the first point of contact for patients with infections and may manage treatment throughout the entire course of illness [7]. For this reason, it is essential that they understand how their prescribing practices can influence the spread of AMR and are equipped with current, evidence-based knowledge on the appropriate use of antibiotics. To support this, the WHO has published the antibiotic book, which offers guidance on the rational use of antibiotics for common infectious conditions, including those frequently encountered in primary care settings [8].

A wide range of international studies has examined antibiotic prescribing practices among GPs. A recent meta-analysis reported that antibiotics are prescribed to 42.1% of patients presenting at the primary care level worldwide, with inappropriate prescribing observed in up to 57.6% of cases [9]. A multi-country survey conducted across several Asian and North African countries found that 41% of GPs considered antibiotics to be indicated for ARI, while a further 27% expressed neutral views [10]. Another meta-analysis demonstrated that early-career GPs prescribe antibiotics less frequently than their more experienced counterparts [11]. Despite the substantial body of international evidence, including multiple meta-analyses, studies from Central Asia remain scarce. Countries in this region share a common healthcare legacy, including similar models of service delivery and system organization [12]. Most available evidence originates from Uzbekistan [13,14], with limited data from other Central Asian countries. To address this gap, the present study aimed to investigate antibiotic resistance awareness and prescribing practices among GPs in Kazakhstan, Kyrgyzstan, Uzbekistan, and Tajikistan.

## 2. Results

The majority of physicians included in the study were female. The median age of participants was 40 years, ranging from 22 to 74 years. Consistent with the level of urbanization in the countries studied, most physicians practiced in urban settings; this proportion was highest in Kazakhstan, where 80.0% of respondents worked in urban areas. The median duration of professional experience was 14.0 years, with physicians from Kyrgyzstan and Uzbekistan reporting longer median work experience (18.5 and 18.0 years, respectively). Most respondents were employed in public healthcare facilities, while employment in private or mixed public–private settings was uncommon across the four countries. The median number of patients seen per day was 20.0; physicians in Uzbekistan reported higher patient volumes (25.0 per day), whereas those in Tajikistan and Kyrgyzstan reported lower volumes (15.0 and 16.0 per day, respectively) (Table 1).

Most physicians in Kazakhstan, Kyrgyzstan, and Uzbekistan perceived the relevance of AMR to their daily practice as moderate, whereas in Tajikistan, the majority of physicians rated its relevance as high (71.4%). Nevertheless, across all countries included in the study, most physicians (71.1% overall) acknowledged that their antibiotic prescribing behavior influences the development of AMR within their regions. The reported frequency of contact with patients carrying multidrug-resistant organisms (MDROs) was generally low. Most physicians in Kazakhstan, Kyrgyzstan, and Uzbekistan described such contact as rare. In contrast, physicians in Tajikistan most frequently reported daily contact with patients with MDROs (31.9%), which may partly explain the higher perceived relevance of AMR in routine clinical practice in this country. When asked which sectors should be targeted to slow the development of AMR, physicians most often selected areas within the human healthcare system, including hospital antibiotic use, general practitioner prescribing, and patient antibiotic consumption. Sectors outside human healthcare were selected less frequently. In Tajikistan, however, antibiotic use in livestock was most frequently identified as a sector that should be targeted. It has to be noted that only a small proportion of respondents across all countries (2.1%) indicated that coordinated action across all sectors is required to effectively address AMR (Table 2).

The strategy of delayed antibiotic prescribing was not known to the largest proportion of physicians, accounting for 27.1% of all responses. Physicians in Uzbekistan reported greater familiarity with this approach, with 26.6% indicating that they use it sometimes and 23.3% stating that they use it often. Patient noncompliance and patient demand for antibiotics were the most frequently reported drivers of unnecessary antibiotic prescribing, at 27.2% and 22.8%, respectively. Acute exacerbation of chronic obstructive pulmonary disease (COPD) with increased volume of purulent sputum and dyspnea was considered the most common indication for antibiotic prescription, selected by 43.5% of physicians. Other indications were also frequently selected, with acute infection with green or yellow sputum reported by 37.4% of physicians overall (Table 3).

Table 4 provides additional insights into factors associated with the correct selection of indications for initiating antibiotic therapy. Among the listed criteria, only acute exacerbation of COPD with increased volume of purulent sputum and dyspnea is a guideline-based indication [8]. Female physicians and older physicians had significantly higher odds of selecting the correct option. Compared with physicians from Kazakhstan, those from Uzbekistan were more likely to choose the correct indication. Physicians practicing in rural areas had lower odds of selecting the correct indication, whereas seeing 20 or more patients per day was associated with higher odds. Among other factors included in the model, only reported use of practice guidelines for antibiotic therapy remained significant, although analysis by frequency of guideline use did not reach statistical significance.

Most physicians reported that they frequently discuss antibiotic resistance with their patients, both when prescribing an antibiotic (35.3%) and when not prescribing one (31.9%). Only 3.9% and 12.3% of physicians, respectively, indicated that they never discuss this issue. The most common reason for not discussing antibiotic resistance was lack of time (43.2%), followed by perceived lack of patient interest (28.3%). Of interest is the fact that physicians in Tajikistan reported that insufficient knowledge of general practitioners on the topic was the main reason for not addressing antibiotic resistance with patients (36.9%) (Table 5).

Overall, most physicians reported frequent use of practice guidelines for antibiotic therapy in their daily work (42.9%), with this proportion being higher in Tajikistan (65.5%). The majority of physicians expressed a desire for more evidence-based therapy guidelines (85.1%). Clinical practice guidelines were the most commonly used source of current information on antibiotic therapy and resistance (20.4% of responses), followed by continuing professional education (15.9%) and textbooks (14.1%). Guidelines also dominated when physicians were asked about additional information sources that would be particularly helpful, with 22.5% indicating a need for better guidelines and 17.7% reporting a need for improved access to existing guidelines. Digital technology-based approaches were less frequently selected as information sources (Table 6).

## 3. Discussion

### 3.1. Physicians’ Knowledge of Antibiotic Resistance and Prescribing Behaviors

The findings of this study provide several important insights and have potential implications for public health policy. Although more than 70% of GPs recognized that their antibiotic prescribing behavior influences the development of AMR in their region, delayed antibiotic prescribing was routinely used by only a small proportion of respondents. In addition, awareness of the One Health approach appeared to be limited, as only 2.1% of participants indicated that interventions across all sectors are necessary to curb the spread of AMR. While antibiotic prescribing rates were not directly assessed, the results suggest a tendency toward antibiotic overuse and misuse. For example, 22.8% of respondents reported that patient demand was a reason for antibiotic prescribing, and 27.2% cited patient noncompliance. The majority of physicians expressed a need for additional information resources to support more rational antibiotic prescribing. Interestingly, digital and technology-based resources were not among the most frequently preferred sources of information. These findings should be interpreted in the context of previous studies on this topic.

An earlier study from Uzbekistan assessed GPs’ knowledge, attitudes, and practices related to AMR among 236 physicians recruited nationwide [13]. In that study, 32.6% of GPs were unaware of the concept of delayed antibiotic prescribing, a proportion higher than that observed in the present study (25.2%). The same study also reported limited awareness of guideline-based prescribing (66.5% of respondents). In contrast, in the present study, 43.9% of Uzbekistani physicians reported frequent use of clinical practice guidelines in their daily work, while an additional 29.5% reported moderate use. Differences were also observed with respect to practice setting. The earlier study concluded that GPs working in rural areas demonstrated relatively higher knowledge levels than their urban colleagues. By contrast, the present study found that employment in an urban setting was a predictor of correctly identifying indications for antibiotic prescribing. It should be noted that many of the discrepancies between the two studies may be attributable to between-sample variability, as both investigations included relatively small samples of GPs in Uzbekistan (236 and 369 participants, respectively).

Another study from Uzbekistan examined antibiotic prescribing practices among rural healthcare physicians and reported a high overall rate of antibiotic prescriptions (56.5%). When stratified by disease group, antibiotics were prescribed to 78.9% of patients presenting with respiratory tract infections and to 93.5% of those with urinary tract infections. It should be noted, however, that this study was published in 2003, and prescribing practices may have evolved substantially since that time [14]. In addition, a multi-country qualitative study that included Tajikistan provided insights into physicians’ decision-making when prescribing antibiotics. The study highlighted patient demand as an important driver of prescribing, particularly requests for injectable formulations, with ciprofloxacin being among the most frequently demanded antibiotics [15].

Ciprofloxacin is classified as a “Watch” group antibiotic under the WHO AWaRe framework, indicating a higher potential for the development of antibiotic resistance [16]. In Central Asian countries, the consumption of “Watch” antibiotics has traditionally been high [17], whereas the use of “Access” antibiotics remains comparatively low, falling short of the WHO target of 60% [18]. Another recurring pattern, also reported in previous studies, is the high proportion of parenteral antibiotic use, observed at both hospital [19] and primary care levels [17]. To some extent, this practice reflects the legacy of the Soviet healthcare model, which placed strong emphasis on hospital-based care and injectable formulations [12]. Public awareness of AMR, together with healthcare-seeking behaviors, further shapes patterns of antibiotic use and contributes to the development and spread of antibiotic resistance [20].

An important observation of this study was that, among the currently used information sources, clinical practice guidelines were the most frequently cited (20.4%). In addition, when asked about unmet needs, 22.5% of respondents indicated a need for higher-quality clinical practice guidelines, while a further 17.7% expressed a desire for better access to existing guidelines. Evidence-based, up-to-date clinical practice guidelines represent the cornerstone of clinical decision-making [21], and it is encouraging that physicians in Central Asia rely on them as a key resource for rational antibiotic prescribing. At the same time, the expressed wish for better access to guidelines, together with the relatively low preference for digital and technology-based information sources observed in this study, suggests that GPs in Central Asia may face technological constraints. Given that the Internet has become a central source of medical information for healthcare professionals worldwide [22], improving digital access and strengthening technological infrastructure may represent an important strategy for supporting evidence-based antibiotic prescribing in the region.

Beyond physicians’ prescribing practices, multiple factors influence antibiotic consumption in the human healthcare sector. An ecological study examining the role of socioeconomic determinants found that mortality rates, birth rates, and under-five mortality were significant drivers of antibiotic consumption in Kazakhstan and Tajikistan, while measles immunization coverage and access to clean cooking fuels were important determinants in Kyrgyzstan [23]. Central Asian countries are characterized by relatively high birth rates, resulting in a large proportion of children within the population [24]. At the same time, vaccine hesitancy has become more prevalent, and recurrent measles outbreaks have been reported, both of which have been associated with increased antibiotic use [25]. As frontline healthcare providers, GPs play an important role in the management of common infectious diseases and are therefore central to initiatives aimed at promoting rational antibiotic use and reducing unnecessary prescribing [26].

An interesting observation of this study is the predominance of female physicians among respondents. This finding is consistent with the historical feminization of the medical profession in Central Asian countries. Earlier research has reported that women constitute the majority of physicians in the region, accounting for approximately 84% of physicians in Kazakhstan and 75% in Kyrgyzstan [27]. This pattern may have important implications for antibiotic prescribing practices. A study from the Netherlands found that female GPs prescribe antibiotics less frequently than their male counterparts when managing patients presenting with sore throat symptoms, particularly when treating female patients. These differences were attributed to variations in communication style and physician–patient interaction [28]. To some extent, this observation may relate to the findings of the present study. Our regression analysis indicated that female sex was associated with better knowledge of appropriate indications for antibiotic prescribing, which could partly explain a potentially lower propensity for unnecessary antibiotic prescribing among female GPs.

Studies on antibiotic resistance in specific pathogens across Central Asia provide important clinical context for this study, although the available evidence remains limited. In Kazakhstan, more than half of *Escherichia coli* isolates and 68% of *Klebsiella pneumoniae* isolates exhibited extended-spectrum beta-lactamase production [29]. Research from Kyrgyzstan has reported high resistance rates among *Neisseria gonorrhoeae* isolates to previously recommended antibiotics, such as ciprofloxacin and tetracycline, while resistance to cefixime, gentamicin, and azithromycin remained rare [30]. In Uzbekistan, enteric pathogens including *Shigella flexneri* and *Shigella sonnei* have shown high resistance rates to commonly used antibiotics, including ampicillin, streptomycin, chloramphenicol, and tetracycline, with multidrug resistance mediated by integrons [31]. Microbiological evidence from other countries in the region, particularly Tajikistan, remains scarce.

### 3.2. Study Limitations

To the best of our knowledge, this is the first study to report on awareness of antibiotic resistance and antibiotic prescribing practices among GPs across several Central Asian countries. Although previous studies have described patterns of antimicrobial consumption in the region [32,33], research examining physicians’ prescribing behavior remains limited. Most of the available evidence comes from studies conducted in Uzbekistan [13,14].

Despite its obvious strengths, this study also has limitations. While the overall sample size of 1231 physicians is sufficient to detect statistically significant differences, it should be acknowledged that the country-specific samples were disproportionate, ranging from 469 GPs in Kazakhstan to 119 GPs in Tajikistan. To some extent, this reflects the smaller population and physician workforce in Tajikistan compared with other Central Asian countries [34].

The pilot sample for the 21-item questionnaire consisted of 20 GPs, which may be considered relatively small. However, pilot studies of this type typically rely on modest samples to assess comprehension and feasibility rather than to perform full psychometric validation. Methodological research indicates that pilot sample sizes of 10–30 participants are generally sufficient to identify major issues in survey instruments [35].

In addition, the study relied on self-reported prescribing behavior without validation against actual prescription data, which introduces the risk of reporting bias. This limitation arises from the multi-country scope of the study and the challenges in accessing standardized prescription records across different healthcare systems.

The Cronbach’s alpha of 0.673 obtained during the questionnaire pilot indicates borderline internal consistency. Nevertheless, since the instrument was designed to assess multiple domains, alpha values in the range of 0.6–0.7 are generally considered acceptable for exploratory instruments measuring diverse constructs [36,37].

Another limitation is the cross-sectional design of the study, which precludes the establishment of causal or robust associative relationships between explanatory factors and knowledge of antibiotic prescribing indications. In addition, the voluntary nature of participation and the reliance on digital communication for survey distribution may have introduced selection bias.

Given these limitations, the findings of this study should be interpreted as exploratory. As one of the first studies to examine antibiotic resistance awareness and prescribing practices among GPs in Central Asia, this work provides a foundation for future research; however, larger and longitudinal studies are needed to confirm these findings and further elucidate the determinants of prescribing behavior in the region.

### 3.3. Conclusions for General Readers

This study examined how general practitioners in Kazakhstan, Kyrgyzstan, Uzbekistan, and Tajikistan understand antibiotic resistance and how they prescribe antibiotics in everyday practice. Most participating physicians worked in public healthcare facilities and practiced in urban areas. They generally recognized that their own prescribing behavior can influence the development of antibiotic resistance in their regions. However, important knowledge gaps were identified. For example, more than one quarter of physicians were unfamiliar with the strategy of delayed antibiotic prescribing, and many reported that patient demand for antibiotics contributes to unnecessary prescribing.

Physicians frequently discuss antibiotic resistance with their patients, although lack of time and perceived lack of patient interest sometimes limit these conversations. Clinical practice guidelines were the most commonly used source of information on antibiotic therapy, and most physicians expressed a strong desire for better guidelines and easier access to them. Overall, the findings suggest that improving access to high-quality clinical guidelines and strengthening professional education could support more appropriate antibiotic prescribing in primary care across Central Asia.

## 4. Materials and Methods

### 4.1. Study Design and Proceedings

To achieve the study aim, the following inclusion criteria were applied: (i) GPs working in one of the countries under study; (ii) current involvement in the provision of patient care, irrespective of healthcare setting (public or private), location (urban or rural), or type of employment contract (part-time or full-time); and (iii) provision of informed consent to participate in the study. The exclusion criteria were: (i) non-GP status; (ii) no current involvement in patient care (e.g., retirement); and (iii) failure to provide informed consent. Prior to study initiation, the research protocol was reviewed and approved by the Institutional Research Ethics Committee of Nazarbayev University (NU IREC), Astana, Kazakhstan (submission No. 851/05022024; approved on 9 April 2024).

The study questionnaire was distributed through professional medical associations of general practitioners between September 2024 and May 2025. The sampling frame consisted of physicians who had registered their mobile telephone numbers with their respective professional associations and had consented to be contacted for research purposes. All physicians meeting the inclusion criteria received an invitation to participate in the online survey, which included a link to the questionnaire administered via the Qualtrics platform. The decision to use mobile telephone numbers for survey distribution was based on the observation that professionals in Central Asia rarely use email for communication and instead predominantly rely on messaging applications such as WhatsApp or Telegram [38]. One month after the initial invitation, a reminder message was sent to encourage participation.

The only personal information collected during the survey was age (in completed years) and sex. No other identifying information, such as names, surnames, or the name of the healthcare facility, was collected. In accordance with IREC requirements, the research team did not have access to participants’ personal contact details; invitations containing survey links were distributed exclusively by representatives of the respective professional medical associations.

### 4.2. Questionnaire

A review panel was convened to identify existing international questionnaires assessing antibiotic resistance awareness and prescribing behavior among general practitioners. Several validated instruments were identified [39,40,41,42,43] and evaluated for their relevance and applicability to the Central Asian context. In selecting the most appropriate questionnaire for the present study, several factors were considered. This is among the first studies to examine GPs’ awareness of the topic in the region, and the primary aim was to gain an understanding of the current situation. In addition, because the study team did not have access to participants’ personal contact details and the questionnaire was administered electronically, brevity was considered essential. Previous research has shown that healthcare professionals are less likely to complete long online surveys, indicating that shorter questionnaires may improve response rates [44]. Taking these considerations into account, the questionnaire developed by Salm et al. [43] was selected. Beyond meeting the above criteria, this instrument uses ARIs as clinical scenarios to assess knowledge of indications for antibiotic prescribing. ARIs are common during the winter months in the countries under study and represent a frequent and well-recognized context for antibiotic prescribing in routine clinical practice [12]. For these reasons, this questionnaire was deemed appropriate for use in the present study.

The questionnaire developed by Salm et al. consists of five thematic blocks: Sociodemographic Data, Relevance, Prescribing Behavior, Communication, and Information Sources. The Relevance block includes four questions assessing physicians’ perceptions of the relevance of antibiotic resistance to their daily clinical practice, the healthcare sector, and other related sectors. The Prescribing Behavior block comprises three questions addressing the use of delayed antibiotic prescribing strategies and clinical indications for antibiotic prescription. The Communication block includes three questions exploring whether and how physicians discuss antibiotic resistance with patients. The Information Sources block consists of four questions examining the use of clinical practice guidelines as well as current and preferred sources of information on antibiotic therapy and antimicrobial resistance [43].

In the subsequent stage, the review panel adapted the Sociodemographic Data block to ensure relevance to the Central Asian context. Specifically, physicians were asked to report: (i) country of practice; (ii) sex; (iii) age; (iv) practice location (urban or rural); (v) years of professional experience; (vi) type of healthcare facility (public, private, or both); and (vii) average number of patients seen per day. The Relevance, Communication, and Information Sources blocks were used without modification. The Prescribing Behavior block was revised to align with the WHO AWaRe Antibiotic Book, released in 2022 [8], after development of the original questionnaire. In particular, the response option “acute exacerbated COPD with a lot of purulent sputum” in the question on indications for antibiotic prescription was refined by adding dyspnea, resulting in the formulation “acute exacerbated COPD with a lot of purulent sputum and dyspnea”. The final version of the questionnaire comprises 21 questions and is provided as a Supplementary File.

Prior to survey administration, the questionnaire was piloted among 20 GPs, and the Cronbach’s alpha coefficient was calculated to assess internal consistency, yielding a value of 0.673. A review panel subsequently evaluated the pilot results and provided feedback, confirming the instrument’s suitability for use in the main study. Responses from the pilot participants were not included in the final analysis.

### 4.3. Data Analysis

Completed questionnaires were downloaded from the Qualtrics platform and exported to Microsoft Excel as value-based tables. The questionnaire was distributed to 2300 physicians, of whom 1563 participated in the survey, resulting in a response rate of 67.95%. These records constituted the initial survey dataset, which was screened for missing values as the first step of data preprocessing. This process identified 332 incomplete responses (21.2%), which were excluded from further analysis. The final analytic dataset included 1231 responses, of which 469 (38.1%) were from Kazakhstan, 274 (22.2%) from Kyrgyzstan, 369 (30.0%) from Uzbekistan, and 119 (9.7%) from Tajikistan.

Normality of distribution for continuous variables was assessed prior to analysis. As the data deviated from a normal distribution, continuous variables were summarized as medians with interquartile ranges (Q1–Q3), and between-group comparisons were performed using the Mann–Whitney U-test. Categorical variables were presented as counts and percentages, with between-group comparisons conducted using Pearson’s chi-square test.

Binary logistic regression analysis was used to examine factors associated with correct responses to the question on indications for antibiotic prescription. As multiple responses were permitted for this question, only respondents who selected “acute exacerbated COPD with a lot of purulent sputum and dyspnea” as the sole option were classified as providing a correct response. All other responses, including selections in combination with this option, were considered incorrect, as they reflected potential antibiotic overuse [8].

Univariate analysis was performed as an initial step to identify variables potentially associated with the outcome. Variables that reached statistical significance in the univariate analysis were subsequently entered into the multivariate logistic regression model to determine independent predictors of a correct response. Backward stepwise elimination using the Wald method was applied. Results are presented as adjusted odds ratios with corresponding 95% confidence intervals and *p*-values.

All statistical analyses were performed using the Statistical Package for the Social Sciences (SPSS), version 24.0. A two-sided *p*-value of 0.05 was considered statistically significant.

## 5. Conclusions

This study provides new insights into antibiotic resistance awareness and prescribing practices among GPs in Kazakhstan, Kyrgyzstan, Uzbekistan, and Tajikistan. Although awareness of antibiotic resistance as a public health threat appears relatively high, challenges related to antibiotic misuse and overuse persist. Given the shared healthcare legacy and similar structural characteristics of healthcare systems in these countries, regionally coordinated interventions may be particularly effective. Strengthening antimicrobial stewardship in primary care (through continuous medical education, improved access to clinical guidelines, and support for ethical prescribing) may contribute to more rational antibiotic use and help reduce the growing burden of antibiotic resistance in the region.

## Figures and Tables

**Table 1:** Demographic characteristics of study participants (n = 1231).

Variables		Total N (%)	Kazakhstan N (%)	Kyrgyzstan N (%)	Uzbekistan N (%)	Tajikistan N (%)	*p*-Value of Pearson’s Chi-Square
Sex	Male	370 (30.1)	162 (34.5)	28 (10.2)	126 (34.1)	54 (45.4)	<0.001
	Female	861 (69.9)	307 (65.5)	246 (89.8)	243 (65.9)	65 (54.6)	
Age, years	Median (Q1; Q3 *)	40.0 (29.0; 54.0)	34.0 (27.0; 47.0)	45.0 (31.0; 57.0)	48.0 (30.0; 56.0)	34.0 (28.0; 45.0)	<0.001 **
	<25	68 (5.5)	42 (9.0)	9 (3.3)	10 (2.7)	7 (5.9)	<0.001
	25–34	435 (35.3)	196 (41.8)	82 (29.9)	104 (28.2)	53 (44.5)	
	35–44	213 (17.3)	90 (19.2)	45 (16.4)	52 (14.1)	26 (21.8)	
	45–54	233 (18.9)	77 (16.4)	49 (17.9)	85 (23.0)	22 (18.5)	
	55–64	233 (18.9)	59 (12.6)	63 (23.0)	100 (27.1)	11 (9.2)	
	≥65	49 (4.0)	5 (1.1)	26 (9.5)	18 (4.9)	0 (0.0)	
Place of work	Urban area	847 (68.8)	377 (80.4)	154 (56.2)	255 (69.1)	61 (51.3)	<0.001
	Rural area	384 (31.2)	92 (19.6)	120 (43.8)	114 (30.9)	58 (48.7)	
Years of work experience	Median (Q1; Q3)	14.0 (3.0; 26.0)	10.0 (2.0; 22.0)	18.5 (5.0; 30.0)	18.0 (4.0; 30.0)	9.0 (3.0; 20.0)	<0.001 **
	<15	636 (51.7)	281 (59.9)	118 (43.1)	161 (43.6)	76 (63.9)	<0.001
	≥15	595 (48.3)	188 (41.1)	156 (56.9)	208 (56.4)	43 (36.1)	
Type of healthcare facility	Public	1057 (85.9)	365 (77.8)	236 (86.1)	343 (93.0)	113 (95.0)	<0.001
	Private	71 (5.8)	42 (9.0)	16 (5.8)	12 (3.3)	1 (0.8)	
	Combined (public + private)	103 (8.4)	62 (13.2)	22 (8.0)	14 (3.8)	5 (4.2)	
Number of patients per day	Median (Q1; Q3)	20.0 (15.0; 30.0)	20.0 (15.0; 30.0)	16.0 (12.0; 23.0)	25.0 (18.0; 33.0)	15.0 (11.0; 20.0)	<0.001 **
	<20	527 (42.8)	177 (37.7)	162 (59.1)	99 (26.8)	89 (74.8)	<0.001
	≥20	704 (57.2)	292 (62.3)	112 (40.9)	270 (73.2)	30 (25.2)	

* Q1, Q3—first, third quartiles; ** test of difference was Mann–Whitney U-test.

**Table 2:** Perceived relevance of antibiotic resistance to clinical practice (n = 1231).

Questions		Total N (%)	Kazakhstan N (%)	Kyrgyzstan N (%)	Uzbekistan N (%)	Tajikistan N (%)	*p*-Value of Pearson’s Chi-Square
Relevance of AB ^®^ resistance to daily work	High	446 (36.2)	160 (34.1)	72 (26.3)	129 (35.0)	85 (71.4)	<0.001
	Moderate	542 (44.0)	233 (49.7)	123 (44.9)	169 (45.8)	17 (14.3)	
	Low	161 (13.1)	43 (9.2)	51 (18.6)	52 (14.1)	15 (12.6)	
	Not at all	82 (6.7)	33 (7.0)	28 (10.2)	19 (5.1)	2 (1.7)	
Does your prescribing behavior influence the AB resistance development within your region?	Yes	875 (71.1)	342 (72.9)	194 (70.8)	261 (70.7)	78 (65.5)	0.034
	No	186 (15.1)	53 (11.3)	42 (15.3)	64 (17.3)	27 (22.7)	
	Don’t know	170 (13.8)	74 (15.8)	38 (13.9)	44 (11.9)	14 (11.8)	
Frequency of contact with patients with MDRO * in daily practice	Daily	242 (19.7)	67 (14.3)	35 (12.8)	102 (27.6)	38 (31.9)	<0.001
	Weekly	222 (18.0)	79 (16.8)	35 (12.8)	98 (26.6)	10 (8.4)	
	Monthly	191 (15.5)	85 (18.1)	36 (13.1)	51 (13.8)	19 (16.0)	
	Rarely	394 (32.0)	170 (36.2)	102 (37.2)	96 (26.0)	26 (21.8)	
	Never	182 (14.8)	68 (14.5)	66 (24.1)	22 (6.0)	26 (21.8)	
Sectors that should be targeted to slower the development of AB resistances (multiple answers were permitted)	Hospital hygiene	143 (5.5)	69 (6.2)	26 (4.8)	36 (5.0)	12 (5.3)	<0.001
	Animal farm hygiene	113 (4.4)	46 (4.2)	34 (6.2)	13 (1.8)	20 (8.8)	
	Private food hygiene	136 (5.2)	62 (5.6)	32 (5.9)	28 (3.9)	14 (6.2)	
	AB use in hospitals	541 (20.8)	242 (21.8)	93 (17.0)	162 (22.7)	44 (19.4)	
	AB prescriptions by GP °	698 (26.9)	280 (25.3)	138 (25.3)	233 (32.6)	47 (20.7)	
	AB intake by patients	743 (28.6)	324 (29.2)	166 (30.4)	220 (30.8)	33 (14.5)	
	AB use in lifestock	221 (8.5)	85 (7.7)	57 (10.4)	22 (3.1)	57 (25.1)	

* MDRO—multidrug-resistant organism; ^®^ AB—antibiotic; ° GP—general practitioner.

**Table 3:** Antibiotic prescribing behavior (n = 1231).

Questions		Total N (%)	Kazakhstan N (%)	Kyrgyzstan N (%)	Uzbekistan N (%)	Tajikistan N (%)	*p*-Value of Pearson’s Chi-Square
Use of the strategy of delayed antibiotic prescribing	Strategy not known	333 (27.1)	123 (26.2)	84 (30.7)	93 (25.2)	33 (27.7)	<0.001
	Very often	107 (8.7)	47 (10.0)	25 (9.1)	29 (7.9)	6 (5.0)	
	Often	232 (18.8)	84 (17.9)	48 (17.5)	86 (23.3)	14 (11.8)	
	Sometimes	235 (19.1)	85 (18.1)	31 (11.3)	98 (26.6)	21 (17.6)	
	Rarely	138 (11.2)	54 (11.5)	26 (9.5)	38 (10.3)	20 (16.8)	
	Never	186 (15.1)	76 (16.2)	60 (21.9)	25 (6.8)	25 (15.1)	
Reasons why antibiotics are prescribed without a hard indication (multiple answers were permitted)	The weekend is approaching, and the course of the disease is difficult to predict	242 (12.5)	123 (14.5)	41 (10.6)	66 (12.7)	12 (6.4)	<0.001
	The patient wants to return to work quickly	266 (13.7)	120 (14.2)	35 (9.1)	60 (11.6)	51 (27.3)	
	The patient demands an antibiotic	442 (22.8)	219 (25.9)	95 (24.7)	99 (19.1)	29 (15.5)	
	The patient is noncompliant	527 (27.2)	210 (24.8)	133 (34.5)	164 (31.6)	20 (10.7)	
	Language barriers or cognitive impairments	36 (1.9)	19 (2.2)	7 (1.8)	8 (1.5)	2 (1.1)	
	The patient is unknown	56 (2.9)	29 (3.4)	10 (2.6)	13 (2.5)	4 (2.1)	
	Further diagnostics are too expensive	153 (7.9)	40 (4.7)	24 (6.2)	71 (13.7)	18 (9.6)	
	To be on the safe side	215 (11.1)	86 (10.2)	40 (10.4)	38 (7.3)	51 (27.3)	
Indications for an antibiotic prescription are for me? (multiple answers were permitted)	Acute infection with white sputum	100 (5.4)	61 (8.6)	10 (2.7)	19 (3.4)	10 (4.8)	<0.001
	Acute infection with green/yellow sputum	691 (37.4)	318 (44.9)	132 (35.9)	174 (31.0)	67 (32.1)	
	Acute exacerbated COPD * with little sputum	252 (13.6)	61 (8.6)	50 (13.6)	105 (18.7)	36 (17.2)	
	Acute exacerbated COPD * with increased volume of purulent sputum and dyspnea	804 (43.5)	269 (37.9)	176 (47.8)	263 (46.9)	96 (45.9)	

* COPD—chronic obstructive pulmonary disease.

**Table 4:** Multivariable logistic regression analysis to determine factors associated with knowledge on antibiotic prescribing indications.

Factors		aOR (95% CI) *	*p*-Value
Sex	Male	Reference	
	Female	1.586 (1.146; 2.195)	0.005
Age, years		1.060 (1.048; 1.072)	<0.001
Country	Kazakhstan	Reference	
	Kyrgyzstan	0.777 (0.440; 1.372)	0.384
	Uzbekistan	2.342 (1.320; 4.155)	0.004
	Tajikistan	1.351 (0.767; 2.378)	0.298
Place of work	Urban area	Reference	
	Rural area	0.710 (0.515; 0.979)	0.037
Number of patients per day	<20	Reference	
	≥20	1.548 (1.145; 2.092)	0.005
Use of practice guidelines for antibiotic therapy	Frequently	Reference	
	Moderately	1.090 (0.544; 2.184)	0.809
	Rarely or never	0.667 (0.324; 1.373)	0.271
	No good guidelines	0.774 (0.380; 1.577)	0.481

aOR (95% CI) *—adjusted odds ratio with 95% confidence interval.

**Table 5:** Communication strategies used by the physicians (n = 1231).

Questions		Total N (%)	Kazakhstan N (%)	Kyrgyzstan N (%)	Uzbekistan N (%)	Tajikistan N (%)	*p*-Value of Pearson’s Chi-Square
Discussion of antibiotic resistance with patients suffering from infections when prescribing an antibiotic	Very often	278 (22.6)	96 (20.5)	69 (25.2)	70 (19.0)	43 (36.1)	0.002
	Often	435 (35.3)	160 (34.1)	106 (38.7)	127 (34.4)	42 (35.3)	
	Partly	281 (22.8)	114 (24.3)	50 (18.2)	101 (27.4)	16 (13.4)	
	Rarely	189 (15.4)	73 (15.6)	41 (15.0)	60 (16.3)	15 (12.6)	
	Never	48 (3.9)	26 (5.5)	8 (2.9)	11 (3.0)	3 (2.5)	
Discussion of antibiotic resistance with patients suffering from infections when not prescribing an antibiotic	Very often	191 (15.5)	77 (16.4)	38 (13.9)	51 (13.8)	25 (21.0)	0.041
	Often	393 (31.9)	130 (27.7)	98 (35.8)	133 (36.0)	32 (26.9)	
	Partly	317 (25.8)	124 (26.4)	72 (26.3)	86 (23.3)	35 (29.4)	
	Rarely	178 (14.5)	77 (16.4)	27 (9.9)	61 (16.5)	13 (10.9)	
	Never	152 (12.3)	61 (13.0)	39 (14.2)	38 (10.3)	14 (11.8)	
Reasons not to talk about antibiotic resistance (multiple answers were permitted)	Lack of time	701 (43.2)	310 (47.8)	147 (43.2)	212 (46.1)	32 (18.2)	<0.001
	Concern to unsettle the patient	174 (10.7)	64 (9.9)	34 (10.0)	29 (6.3)	47 (26.7)	
	Lack of patient interest	459 (28.3)	177 (27.3)	114 (33.5)	136 (29.6)	32 (18.2)	
	Lack of GP’s ° knowledge about the subject	290 (17.9)	97 (15.0)	45 (13.2)	83 (18.0)	65 (36.9)	

° GP—general practitioner.

**Table 6:** Sources of information on antibiotic therapy and antimicrobial resistance (n = 1231).

Questions		Total N (%)	Kazakhstan N (%)	Kyrgyzstan N (%)	Uzbekistan N (%)	Tajikistan N (%)	*p*-Value of Pearson’s Chi-Square
Use of practice guidelines for antibiotic therapy during daily work	Frequently	528 (42.9)	174 (37.1)	114 (41.6)	162 (43.9)	78 (65.5)	<0.001
	Moderately	306 (24.9)	118 (25.2)	62 (22.6)	109 (29.5)	17 (14.3)	
	Rarely or never	343 (27.9)	145 (30.9)	90 (32.8)	88 (23.8)	20 (16.8)	
	There are no good guidelines	54 (4.4)	32 (6.8)	8 (2.9)	10 (2.7)	4 (3.4)	
Desire for more evidence-based therapy guidelines	Yes	1047 (85.1)	383 (81.7)	225 (82.1)	341 (92.4)	98 (82.4)	<0.001
	No	82 (6.7)	35 (7.5)	21 (7.7)	13 (3.5)	13 (10.9)	
	Don’t know	102 (8.3)	51 (10.9)	28 (10.2)	15 (4.1)	8 (6.7)	
Sources of current information on antibiotic therapy and resistance (multiple answers were permitted)	Internet forums	224 (7.8)	98 (7.9)	50 (8.6)	64 (7.4)	12 (6.0)	<0.001
	Digital information platforms	397 (13.8)	223 (18.1)	49 (8.4)	96 (11.1)	29 (14.5)	
	Textbooks	405 (14.1)	176 (14.3)	69 (11.9)	138 (16.0)	22 (11.0)	
	Scientific journals	323 (11.2)	171 (13.9)	47 (8.1)	85 (9.8)	20 (10.0)	
	Clinical practice guidelines	586 (20.4)	250 (20.3)	145 (24.9)	166 (19.2)	25 (12.5)	
	Direct communication with colleagues	239 (8.3)	90 (7.3)	41 (7.0)	77 (8.9)	31 (15.5)	
	Direct communication with an expert	248 (8.6)	112 (9.1)	32 (5.5)	70 (8.1)	34 (17.0)	
	Continuing education	457 (15.9)	114 (9.2)	149 (25.6)	167 (19.4)	27 (13.5)	
Additional information sources that would be helpful (multiple answers were permitted)	No further sources needed	161 (5.6)	77 (6.4)	31 (5.0)	29 (3.5)	24 (10.1)	<0.001
	Interdisciplinary network	213 (7.4)	119 (9.9)	32 (5.2)	37 (4.5)	25 (10.5)	
	Better clinical practice guidelines	646 (22.5)	253 (21.0)	126 (20.5)	216 (26.3)	51 (21.4)	
	Better access to existing guidelines	509 (17.7)	195 (16.2)	150 (24.4)	140 (17.1)	24 (10.1)	
	Interactive case studies	91 (3.2)	42 (3.5)	14 (2.3)	26 (3.2)	9 (3.8)	
	Training games	306 (10.6)	129 (10.7)	71 (11.5)	84 (10.2)	22 (9.2)	
	Information and training App	260 (9.0)	102 (8.5)	46 (7.5)	86 (10.5)	26 (10.9)	
	Webpage with news and links	401 (13.9)	173 (14.4)	71 (11.5)	115 (14.0)	42 (17.6)	
	E-learning based trainings	290 (10.1)	113 (9.4)	74 (12.0)	88 (10.7)	15 (6.3)	

## Data Availability

The data presented in this study are available on reasonable request from the corresponding author.

## Supplementary Materials

The following supporting information can be downloaded at https://www.mdpi.com/article/10.3390/antibiotics15030309/s1, Survey Questionnaire.

## Abbreviations

The following abbreviations are used in this manuscript:

AB	Antibiotic
AMR	Antimicrobial Resistance
AMS	Antimicrobial Stewardship
aOR	Adjusted Odds Ratio
ARI	Acute Respiratory Infection
AWaRe	Access, Watch, Reserve
COPD	Chronic Obstructive Pulmonary Disease
GP	General Practitioner
MDRO	Multidrug-Resistant Organism
OTC	Over-the-Counter
SPSS	Statistical Package for the Social Sciences
WHO	World Health Organization
95% CI	95% Confidence Interval
