# Supplementary material for: Antibiotic Resistance Awareness and Prescribing Behavior Among General Practitioners in Kazakhstan, Kyrgyzstan, Uzbekistan, and Tajikistan
Source: Antibiotics (Basel). 2026 Mar 18;15(3):309. doi: 10.3390/antibiotics15030309 (PMC13024538; doi:10.3390/antibiotics15030309)
Supplement: Supplementary file 1 [file antibiotics-15-00309-s001.zip › antibiotics-4168041-supplementary.pdf]

Survey Questionnaire

1. **Sociodemographic data**

1. Please indicate your gender:
   1. Male
   2. Female

2. Please indicate your age in years

3. Please indicate your country of residence***:***
   1. Kazakhstan
   2. Kyrgyzstan
   3. Uzbekistan
   4. Tajikistan

4. Please indicate where you work:
   1. Urban area
   2. Rural area

5. Please indicate your years of work experience

6. Please select the type of clinic where you practice:
   1. Public healthcare facility
   2. Private healthcare facility
   3. Combined practice (public + private)

7. Please indicate the number of patients you see in a day:

3. **Relevance**

1. How relevant is the subject of antibiotic resistance for your daily work?

highly/ moderately/ sparsely/ not at all

2 Do you think, that your antibiotic prescribing behavior influences the antibiotic resistance development within your region?

yes/ no/ don't know

3. How often do you have contact to patients with MDRO during your daily practice?

daily/ weekly/ monthly/ rarer/ never

4 What do you think, which sectors should be targeted to slower the development of antibiotic resistances?

- Hospital hygiene
- Animal farm hygiene

- Private food hygiene
- AB use in hospitals
- AB prescriptions by GP
- AB intake by patients
- AB use in lifestock

4. **<u>Prescribing behavior</u>**

5. Do you use the strategy of delayed antibiotic prescribing?
Strategy not known/ very often/ often/ sometimes/ rarely/ never

6. What are the reasons why antibiotics are prescribed without a hard indication?
Multiple selection:

- when the weekend is approaching and the course of the disease is difficult to predict
- if the patient wants to get back to work quickly
- if the patient demands an antibiotic
- if the patient is incompliant
- language barriers or cognitive impairments
- unknown patient
- because further diagnostics are too expensive
- to be on the safe side

7. Indications for an antibiotic prescription are for me*?*
Multiple selection:

- acute infection with white sputum
- acute infection with green/yellow sputum
- acute exacerbated COPD with little sputum
- acute exacerbated COPD with a lot of purulent sputum

5. **<u>Communication</u>**

***8a.* :** Do you discuss the subject of AMR with your patients suffering from infections?

… while prescribing an antibiotic:

very often/ often/ partly/ rarely/ never

8b. Do you discuss the subject of AMR with your patients suffering from infections?

… while not prescribing an antibiotic:

very often/ often/ partly/ rarely/ never

9. Reasons not to talk about antibiotic resistance (AMR).

Multiple selection:

- Lack of time
- Concern to unsettle the patient
- Lack of patient interest
- Lack of GP´s knowledge about the subject

6. Sources of information

10. Do you use practice guidelines for antibiotic therapy during your daily work?
frequently/ moderately/ rarely or never/ there are no good guidelines

11. Would you like to have more evidence-based therapy guidelines?
yes/ no/ don't know

12. Which are your sources to get current information on antibiotic therapy and AMR?
Multiple selection:

- Internet forums
- digital information platforms
- textbooks
- scientific journals
- Clinical practice guidelines
- direct communication with peer collegues
- direct communication with expert
- continuing education

13. Which additional information sources would be particularly helpful?
Multiple selection:

- No further sources needed, existing ones are sufficient
- interdisciplinary network
- better clinical practice guidelines
- better access to existing guidelines
- interactive case studies
- training games
- information and training App
- Webpage with news and links
- more continuing education without industry sponsoring
- e-learning based trainings
